# The effects of bright light treatment on affective symptoms in people with dementia: a 24-week cluster randomized controlled trial

**DOI:** 10.1186/s12888-021-03376-y

**Published:** 2021-07-28

**Authors:** Eirin Kolberg, Gunnhild Johnsen Hjetland, Eirunn Thun, Ståle Pallesen, Inger Hilde Nordhus, Bettina S. Husebo, Elisabeth Flo-Groeneboom

**Affiliations:** 1 Department of Clinical Psychology, Faculty of Psychology, University of Bergen (UiB), Aarstadveien 17, 5009 Bergen, Norway; 2 City Department of Health and Care, City of Bergen, Norway; 3 Department of Psychosocial Science, Faculty of Psychology, University of Bergen (UiB) Christies gate 12, 5015 Bergen, Bergen, Norway; 4 Norwegian Competence Center for Sleep Disorders, Haukeland University Hospital, Bergen, Norway; 5 Optentia, the Vaal Triangle Campus of the North-West University, Vanderbijlpark, South Africa; 6 Department of Behavioral Medicine, Faculty of Medicine, University of Oslo, (UiO), Oslo, Norway; 7 Centre for Elderly and Nursing Home Medicine (SEFAS), Department of Global Public Health and Primary Care, University of Bergen (UiB), Aarstadveien 17, 5009 Bergen, Norway

**Keywords:** Dementia, Nursing homes, Bright light therapy, Depression, Affective symptoms, Behavioral and psychological symptoms of dementia, Clinical trial

## Abstract

**Background:**

The majority of people with dementia have behavioral and psychological symptoms of dementia (BPSD), including depression, anxiety and agitation. These may be elicited or aggravated by disrupted circadian rhythms. Bright light treatment (BLT) is a promising non-pharmacological approach to the management of BPSD, but previous research has yielded mixed results.

**Methods:**

Eight nursing home dementia units (1 unit = 1 cluster) with 78 patients were invited to participate in a cluster randomized controlled trial from September 2017 to April 2018 investigating the effects of BLT on sleep and circadian rhythms (primary outcome) and BPSD (secondary outcome). Ceiling mounted LED-panels were installed in the intervention group (four units), providing light at 1000 lx and 6000 K (vertically at 1.2 m) between 10 a.m. and 3 p.m., with lower values in the mornings and evenings. Standard indoor light was used in the control group (four units). BPSD were assessed with The Cornell Scale for Depression in Dementia (CSDD) and the Neuropsychiatric Inventory Nursing Home Version (NPI-NH). Data collection took place at baseline and after 8, 16 and 24 weeks. Multilevel regression models with and without false discovery rate correction were used for the analysis, with baseline values and dementia stage entered as covariates.

**Results:**

Sixty-nine patients were included in the study at baseline. Compared to the control group, the intervention group had a larger reduction on the composite scores of both the CSDD (95% CI = − 6.0 – − 0.3) and the NPI-NH (95% CI = − 2.2 – − 0.1), as well as on the NPI-NH Affect sub-syndrome, and the CSDD Mood related signs sub-scale at follow-up after 16 weeks. With FDR correction, the group difference was significant on the CSDD Mood related signs sub-scale (95% CI = − 2.7 – − 0.8) and the NPI-NH Affect sub-syndrome (95% CI = − 1.6 – − 0.2). No differences were found between conditions at weeks 8 or 24.

**Conclusion:**

Compared to the control condition, affective symptoms were reduced after 16 weeks in the group receiving BLT, suggesting BLT may be beneficial for nursing home patients with dementia.

**Trial registration:**

ClinicalTrials.gov Identifier: NCT03357328. Retrospectively registered on November 29, 2017.

## Background

Behavioral and psychological symptoms of dementia (BPSD), including depression, anxiety, agitation and sleep problems have significant impact on the quality of life and care requirements of nursing home patients. The treatment of these symptoms can be challenging and complex [1, 2]. Pharmacotherapy is widely used in the management of depression and other BPSD, despite mixed evidence regarding efficacy and a high risk of adverse outcomes, including mortality [3–6]. Environmental and behavioral interventions are recommended as a first-line of treatment, but are often underutilized as they are challenging to implement, require time, staff resources and training, and may have limited efficacy in acute situations [7]. Research suggests that bright light treatment (BLT) represents a feasible non-pharmacological intervention, with studies reporting improvements in agitation, depression and sleep for people with dementia [8–10].

There are multiple mechanisms that may explain how light affects BPSD. Light plays a key role in regulating circadian rhythms [11, 12], which entail 24-h cycles in the activity of most bodily processes, including sleep-wake behavior, hormone secretion, metabolism and immune functions [13, 14], which are essential to health and well-being. Circadian rhythms are orchestrated by the suprachiasmatic nucleus of the hypothalamus [15], and entrained mainly by retinal illuminance [12]. Light of short wavelengths (i.e., high correlated color temperatures, CCT) and/or high illuminance (light intensity) is the most effective at eliciting non-visual responses such as circadian entrainment [16, 17].

Disruption of circadian rhythms has been implicated as a contributing factor to a range of health problems [18], mood disorders [19], sleep disturbances [20], and even neuropsychiatric disorders such as dementia [21]. It is well-established that sleep is related to mood and mental health [22]. Disturbed circadian rhythms and sleep may thus represent an important pathway through which light affects mood.

Light may also have acute and direct effects on mood, alertness, and cognitive function though pathways from the retina (e.g., to hypothalamic and limbic regions) that do not depend on the suprachiasmatic nucleus [11, 23, 24]. BLT is recommended as the treatment of choice for seasonal affective disorder [25, 26], and multiple studies have found evidence that BLT may improve depression in non-seasonal affective disorders [27–29], also in older adults [29, 30].

Providing BLT to people with dementia by using light boxes can be challenging, as they require patients to remain in front of the light source for the duration of the treatment. Using ceiling-mounted light technology allows for delivery of BLT without interfering with the daily routine at nursing homes, as all patients can receive BLT simultaneously, without staff facilitation.

In one of the few studies to date on ambient BLT in dementia units, Riemersma-van der Lek et al. [31] found that ceiling mounted whole-day light treatment (±1000 lx) significantly ameliorated depressive symptoms (measured by the Cornell Scale for Depression in Dementia, CSDD) in a double-blind trial (*n* = 189). Depression scores were reduced by 1.47 points, or 19%, after 3.5 years (1.76 points at a 1.5-year follow-up), in the group receiving BLT alone (*n* = 49) [31]. Using a pre-post design (*n* = 14 nursing home patients), Figueiro et al. found that 300–400 lx of high-CCT all-day ambient light improved depression, agitation, and sleep after 4 weeks [10]. Other studies on ambient BLT for dementia patients have, however, reported conflicting or mixed results, possibly due to significant methodological differences [32, 33].

Systematic reviews of research on BLT in dementia have called for more high-quality research and detailed reporting of procedures in order to determine the appropriate intensity, frequency, method of delivery, duration, and timing of light treatment on outcomes [34–37]. In addition, the dementia population is heterogenous, and the efficacy of BLT may depend on the severity of the disease [38, 39]. Few studies investigating the effect of BLT on depression have lasted for more than 4 weeks [29, 35, 40]. We aimed to take these concerns into account by controlling for dementia severity, reporting detailed information about light parameters measured at eye level, and conducting a trial of long duration with data collection at multiple points, in order to ascertain the time needed to achieve any beneficial effect.

The present results are secondary outcomes from the 24-week cluster randomized controlled DEM.LIGHT trial. In the present study, the main aim was to assess whether BPSD, as measured by the Neuropsychiatric Inventory Nursing Home Version (NPI-NH) and the Cornell Scale for Depression in Dementia (CSDD), were reduced from baseline to follow up at weeks 8, 16 and 24 in the group receiving BLT compared to the control group. In order to gain better understanding of the results, the correlations between the outcome scales at baseline were also investigated. Our hypothesis was that BPSD would be reduced in the group receiving BLT compared to the control group at follow-ups.

## Methods

### Trial design

The DEM.LIGHT trial (“*Treatment Light Rooms for Nursing Home Patients with Dementia– Designing Diurnal conditions for Improved Sleep, Mood and Behavioral Problems*”, ClinicalTrials.gov Identifier: NCT03357328) was a cluster randomized placebo-controlled trial conducted from September 2017 to April 2018 in Bergen, Norway. Data was collected at four time points; at baseline, week 8, week 16 and week 24. The data collected included proxy-rated questionnaires about BPSD, sleep, activities of daily living, quality of life, and resource utilization; a pain assessment; cognitive assessment; information from medical journals; and assessment of sleep and circadian rhythms. Sleep outcomes have been reported previously [41]. The study adheres to the CONSORT guidelines [42].

### Participants

All nursing homes with a dedicated dementia unit in Bergen municipality, Norway were eligible unless they were participating in other projects or had architectural features prohibiting installation of the light panels. Out of 14 invited nursing home unit leaders, 8 agreed to partake in the trial, and we thus invited a total of 78 residents to participate. The units that were not included either declined to participate (four units), were excluded due to having twice as many residents as other units (one unit), or signalled interest only after the desired number of units was achieved (one unit). All residents at the participating units were screened for inclusion (see Table 1 for eligibility criteria) by clinical psychologists (EK and GJH), in collaboration with the nursing home physician. Inclusion criteria were that the patients had to be at least 60 years old; be in long term (i.e., > 4 weeks) care; have dementia according to the DSM-5 criteria; have sleep/circadian rhythm disturbance, BPSD, or reduced activities of daily living (ADL); and that consent was given for participation by the patient or a proxy. Patients were excluded if they were blind; unable to benefit from BLT; were already taking part in another trial; had a condition contra-indicated to the intervention; had an advanced, severe medical disease and/or expected survival of less than 6 months, or other aspects that could interfere with participation; and if they were psychotic or had a severe mental disorder. Legal guardians provided consent on behalf of the patients after receiving information verbally and in writing. Patients who were potentially able to understand were informed in a personally adapted manner, and given the option to not consent. Verbal and non-verbal expressions of unwillingness to participate by the patients were regarded as withdrawal of consent during the whole data collection. Patients were also allowed freely to withdraw to other areas if they were uncomfortable with the light. Recruitment of nursing home units and patients took place between September 2016 and August 2017, ensuring that participants had spent at least 1 month in the nursing home unit before baseline measurements. Resident physicians and nursing home staff were encouraged to provide care as usual, including necessary psychopharmacological treatment.

**Table 1:** Study inclusion and exclusion criteria

Participants were eligible if they:	Patients were not eligible if they:
- were ≥ 60 years and in long-term care (> 4 weeks) - had dementia in accordance with DSM-5 - had either sleep/circadian rhythm disturbances, BPSD as identified by NPI-NH, or severely reduced ADL function - provided written informed consent if the participant had capacity or, if not, a written proxy informed consent from a legally authorized representative	- were blind or might otherwise not benefit from light - took part in another trial - had a condition contra-indicated to the intervention - had an advanced, severe medical disease/disorder and/or expected survival of less than 6 months, or other aspects that could interfere with participation - were psychotic or had a severe mental disorder

*ADL* Activities of Daily Living, *BPSD* Behavioral and Psychological Symptoms of Dementia, *DSM-5* Diagnostic and Statistical Manual of Mental Disorders-5, *NPI-NH* Neuropsychiatric Inventory-Nursing Home Version

#### Group allocation and blinding

Eight nursing homes were randomized (one cluster per nursing home) by EK and EF to either the intervention group (four clusters) or the control group (four clusters), using random group assignment in SPSS [43]. All participants in each nursing home unit were thus assigned to the same group. Employees in the nursing home units were only told that the researchers were investigating the effect of different kinds of light, not specifically which aspects of the light they would be studying. Blinding of residents was not considered an issue due to the degree of memory loss experienced by those in the target population.

#### Delivery of the intervention

**Fig. 1:**
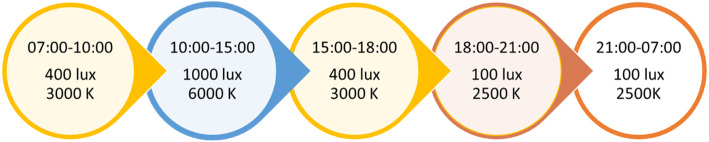
Phases of the light sequence in the intervention group. Illuminance (lux) and correlated color temperature (kelvin, K) at different times of the day in the intervention group, with gradual transition periods of 30 min separating each phase. Between 21:00 and 07:00 o’clock the lights could also be turned off by staff if this was preferred

Ceiling mounted LED light panels (Glamox, 1 x C95 48 CCT 6500 K MP 47 W/4702 lm) were installed in the living rooms of the four nursing home units in the intervention group. The number of light panels needed to provide the required illuminance was calculated for each site by Glamox engineers, taking room size and number of windows into account. The lights were programmed to deliver light at varying illuminances and CCT throughout the day with gradual transition periods, mimicking daily variations in the natural light cycle (see Fig. 1). Peak illuminance and color temperature was delivered between 10 a.m. and 3 p.m. each day, and consisted of approximately 1000 lx and 6000 K (vertically) at the cornea (falling within the interquartile range of observed CCT values for natural daylight across atmospheric conditions, i.e. 5712–7757 K [44]). In the nursing home units assigned to the control group, lights were also changed, but the new light bulbs (CFL AURA UNIQUE-D/E LL 18 W/830 G241–2 in three units and CFL AURA UNIQUE-L LL 18 W/830 2G11 in one) delivered standard indoor illumination (~ 3000 K, 150–300 lx at eye level in the center of the room). In addition to measurements taken by engineers during the installation, illuminance was measured after the start of the trial in all eight units, using the GL Spectis 1.0 T Flicker spectrometer (GL Optic). Measurements were taken vertically at 1.2 m above the floor, to approximate corneal illuminance for a seated patient. Melanopic equivalent daylight (D65) illuminance (EDI) was calculated according to recommendations by the International Commission on Illumination (CIE) [45], using the CIE S 026 toolbox [46]. The daily schedules of the patients were not altered to encourage exposure. Rather, the time spent in the intervention area was meant to reflect the regular habits of the patients.

#### Outcomes

The aim of this study was to investigate the effect of BLT on BPSD. In addition to the BPSD-measures described below, demographic information and health data were extracted from patients’ medical journals by authors with clinical authorization. Additionally, the Mini-Mental State Examination (MMSE) was administered by clinical psychologists (EK and GJH). The MMSE is a validated brief clinician-administered test of cognitive functions, such as orientation, reading, writing, and memory, scored on a scale with a composite score ranging from 0 to 30 where a lower score indicates more impaired cognition [47, 48]. The Charlson Comorbidity Index (CCI) was completed by the researchers based on the patients’ medical journal. The CCI is a tool for classifying comorbid conditions, with weights assigned according to the number and seriousness of diseases. A higher score is associated with increased 1-year mortality rates [49]. The Functional Assessment Staging Test (FAST) describes seven stages in the progression of Alzheimer’s disease, with good validity and reliability [50, 51]. It focuses on the ability to perform activities of daily living, delineating the progressive loss of functioning through seven stages (from 1 = normal adult to 7 = severe Alzheimer’s).

Symptoms of BPSD were evaluated at baseline and after 8, 16 and 24 weeks, using proxy-rater scales validated for people with dementia. See Table 2 for an overview of items contained in the scales and sub-scales used. Proxy-raters were nursing home staff that knew the patients well. Questionnaires were completed with guidance from researchers to ensure consistency of completion across different nursing home units.

**Table 2:** Overview of outcome scales and sub-syndromes included in analyses

CSDD total	Mood-related signs, behavioral disturbance, cyclic functions (see descriptions below), physical signs (loss of appetite, weight loss, and loss of energy), and ideational disturbance (suicidal ideation, low self-esteem, pessimism, and mood-congruent delusions).
CSDD Mood-related signs	Anxiety, sadness, lack of reactivity to pleasant events, and irritability.
CSDD Behavioral disturbance	Agitation, psychomotor retardation, multiple physical complaints, and loss of interest.
CSDD Cyclic functions	Diurnal variation (mood worse in the morning), difficulty falling asleep, multiple nocturnal awakenings, and early-morning awakening.
NPI-NH total	Delusions, hallucinations, dysphoria, anxiety, agitation/aggression, euphoria, disinhibition, irritability/lability, apathy, aberrant motor activity, sleep and night-time behavior, and appetite and eating.
NPI-NH Agitation	Agitation/aggression, disinhibition and irritability.
NPI-NH Affective symptoms	Depression and anxiety.
NPI-NH Psychosis	Delusions and hallucination.

*CSDD* The Cornell Scale for Depression in Dementia, *NPI-NH* The Neuropsychiatric Inventory Nursing Home Version

***The Cornell Scale for Depression in Dementia (CSDD)*** [52] consists of 19 items, each reflecting the presence of an observable symptom in the preceding week. The items are rated as absent (0), mild/intermittent [1] or severe [2], resulting in a composite score ranging from 0 to 38. Individual items are clustered in groups of five sub-scales under the headings “Mood-related signs” (consisting of anxiety, sadness, lack of reactivity to pleasant events, and irritability), “Behavioral disturbance” (consisting of agitation, psychomotor retardation, multiple physical complaints, and loss of interest), “Cyclic functions” (mood worse in the morning, difficulty falling asleep, multiple nocturnal awakenings, and early-morning awakening), “Physical signs” (loss of appetite, weight loss, and loss of energy), and “Ideational disturbance” (suicidal ideation, low self-esteem, pessimism and mood-congruent delusions). Sub-scale scores (range 0–8) were calculated for the first three of these sub-scales. Physical signs were excluded based on the high occurrence of severe somatic illness in the sample, making it difficult to identify physical signs as depressive symptoms. Ideational disturbance was excluded because few of the participants were capable of verbally expressing such ideas. The CSDD has shown high interrater reliability, internal consistency and sensitivity [52], and the Norwegian translation has demonstrated satisfactory psychometric properties [53].

***The Neuropsychiatric Inventory Nursing Home Version (NPI-NH)*** assesses 12 common psychological and behavioral symptoms in dementia: delusions, hallucinations, dysphoria, anxiety, agitation/aggression, euphoria, disinhibition, irritability/lability, apathy, aberrant motor activity, sleep and night-time behavior, and appetite and eating [54]. Each symptom is scored according to frequency (range 0–4) and severity (range 0–3) of the symptoms in the preceding week. The product of the scores of each item (frequency x severity, range 0–12) are added up to a total score (range 0–144). We also report scores on the sub-syndromes “Affective symptoms” (depression and anxiety, range: 0–24), “Psychosis” (delusions and hallucination, range: 0–24), and “Agitation” (consisting of items on agitation/aggression, disinhibition and irritability, range: 0–24), which are based on stable co-occurrence of symptoms in factor analyses [55]. The Norwegian version of the NPI-NH has good reliability and validity [56].

***Estimated light exposure time*** was assessed by asking staff to estimate how many hours the patient on average had spent in the living room between 10 a.m. and 3 p.m. (i.e., the period of peak illuminance and CCT in the intervention condition) since the last data collection.

***Other measurements used in baseline correlations.*** The Sleep Disorder Inventory (SDI) is an extension of the NPI-NH, and was scored by summarizing the severity x frequency ratings for seven symptoms (range 0–84) [41, 57]. The SDI has been shown to correspond well with actigraphy-measured sleep in people with dementia [57]. Wake After Sleep Onset (WASO) was assessed using actigraphs (*Actiwatch II, Philips Respironics)* worn on the dominant wrist [58] for 1 week, during the same week as the questionnaire completion took place or in the week preceding it. Medium sensitivity, and an epoch length of 1 min. Were used. Due to absent cues for accurately determining rest intervals, fixed intervals were set for the rest period (10 p.m. to 6 a.m.), and these intervals were then automatically analysed by the Actiware software *(version 6.0.9, Philips Respironics)* to yield the number of minutes spent awake or asleep in each interval. WASO was defined as the number of minutes spent awake between the onset of the first sleep period and the final awakening in the rest interval. WASO was chosen because it is less impacted than sleep onset latency and early morning awakening when using fixed rest intervals.

#### Sample size and power calculation

The necessary sample size was estimated with an expectation of moderate effect sizes (Cohen’s d = .50) using ANOVA analysis. With a .05 alpha level (two-tailed), and the power set to .80, the power-analysis indicated that a minimum of 64 participants and 8 clusters were needed in order to detect differences between active and control conditions [59, 60]. The aim was to recruit 80 participants, allowing for a 20% dropout.

#### Data management and statistical analyses

Statistical analyses were conducted using R [61]. For all outcomes, multilevel regression models were fitted with lme4 [62] using restricted maximum likelihood estimation, and with a random intercept for each patient. As the residuals for models using untransformed NPI-NH total and sub-syndrome scores violated distributional assumptions, a square root transformation (with an added constant of 0.001) were applied to all NPI-NH scores. After transformation of NPI-NH scores, all models satisfied assumptions of multilevel linear regression modelling. Estimated marginal means with confidence intervals were calculated for all outcomes. The NPI-NH scores were calculated from transformed variables, and then back-transformed for estimated marginal means. All models were fitted with and without a Benjamini-Hochberg [63] false discovery rate (FDR) correction, which adjusts the significance level to account for an increased probability of a type 1 error when multiple tests are conducted. Both corrected and uncorrected results are reported [64]. Associations between variables at baseline were investigated using Spearman correlations.

The FAST score was added as a predetermined covariate to all analyses in order to control for dementia severity, following recommendations by previous authors [34]. Baseline levels of the dependent variable were also added as a covariate to all models [65]. In addition, the following covariates were tested after the completion of the main analysis to investigate their impact on the results: time spent in the living room (i.e., exposure time in the intervention group), having an Alzheimer’s diagnosis, age, the number of psychotropic medications prescribed for regular use, melanopic EDI, prescription of sedatives or hypnotics, being diagnosed with an eye disease, and scores on the Charlson Comorbidity Index. The sample size was not sufficient to perform sub-group analyses on categorization based upon the aforementioned or other variables, such as dementia sub-type.

Patients missing 20% or more of a single outcome scale at any time point were excluded from analysis at that particular time point. If less than 20% was missing, data points were imputed using expectation maximization in SPSS [43]. Patients in either group who had spent less than 30 min on average per day in the living room since the previous data collection were excluded from analysis.

## Results

**Fig. 2:**
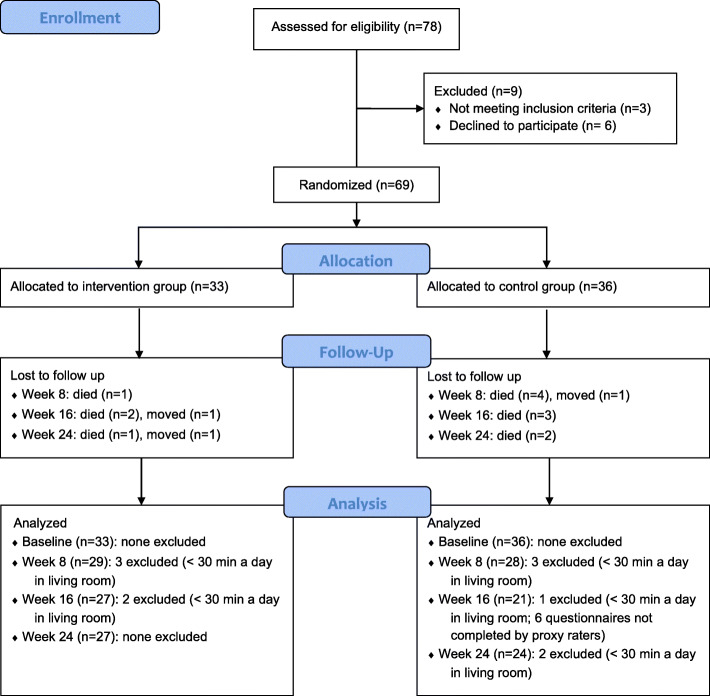
Participant flow

**Fig. 3:**
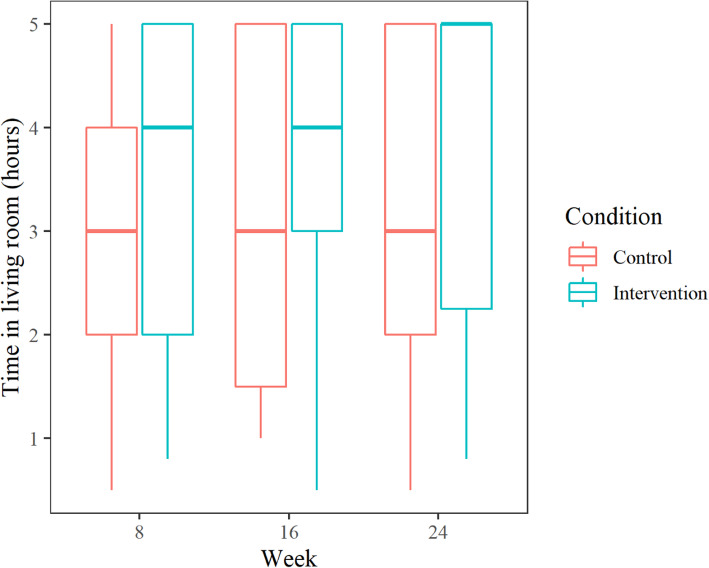
Time spent in living room (daily average) between 10 a.m. and 3 p.m.* since the previous data collection. *Corresponding to the period of peak illumination (1000 lx and 6000 K) for the intervention group. Horizontal line = median, boxes = 25. – 75. percentiles, ends of whiskers = min / max. After exclusion of patients who spent < 30 min in the living room in this time period

Sixty-nine patients from eight dementia units at separate nursing homes were included, out of 78 potential participants. Reasons for exclusion were failure to meet eligibility criteria (*n* = 3) and declining to participate (*n* = 6). Figure 2 shows a flow diagram detailing the number of patients included at each stage of the trial, as well as reasons for those excluded. In all, 38 of the included patients (55%) had an Alzheimer’s diagnosis, 21 (30.5%) had an unknown dementia type, and 7 (10%) had other dementia diagnoses. Three patients did not have a registered dementia diagnosis but were included based on an MMSE score below 26 and assessment by clinicians. Two of the included patients were diagnosed with Parkinson’s disease. Baseline descriptive statistics are reported in Table 3. The median age of the participants at baseline was 85 years, 47 patients (68%) were female, and the median MMSE score was 4, corresponding to severe cognitive impairment [48]. The estimated time per day that each group spent in the living room between 10 a.m. and 3 p.m. is shown in Fig. 3. The types of psychotropic medications prescribed for regular use at each time point are shown in Table 4. Baseline scores on outcome scales in the intervention and control groups are presented in Table 5. Notably, the intervention group median on the CSDD was 11, while the control group median was 6. On the NPI-NH, the intervention group median was 24, whereas the control group median was 12.5 (Table 5).

**Table 3:** Baseline descriptive statistics

	Control (*N* = 36)	Intervention (*N* = 33)	Total (*N* = 69)
Gender			
*Female*	22 (61.1%)	25 (75.8%)	47 (68.1%)
*Male*	14 (38.9%)	8 (24.2%)	22 (31.9%)
Age			
*Median (Q1, Q3)*	82.5 (77.5, 88.0)	86.0 (83.0, 88.0)	85.0 (79.0, 88.0)
FAST			
*Missing*	1	1	2
*4*	1 (2.9%)	2 (6.2%)	3 (4.5%)
*5*	1 (2.9%)	2 (6.2%)	3 (4.5%)
*6*	24 (68.6%)	25 (78.1%)	49 (73.1%)
*7*	9 (25.7%)	3 (9.4%)	12 (17.9%)
Charlson			
*Median (Q1, Q3)*	1.0 (1.0, 2.0)	2.0 (1.0, 2.0)	1.0 (1.0, 2.0)
MMSE			
*Missing*	6	3	9
*Median (Q1, Q3)*	3.0 (1.0, 6.8)	6.0 (2.0, 10.0)	4.0 (1.0, 9.2)
No. of psychotropic drugs			
*Mean (range)*	2.91 (1–6)	2.78 (0–5)	2.85 (0–6)
SDI			
*Median (Q1, Q3)*	3.0 (0.0, 18.0)	3.0 (0.0, 12.5)	3.0 (0.0, 17.5)
WASO			
*Median (Q1, Q3)*	73.9 (34.8, 106.6)	56.5 (32.9, 85.2)	62.7 (32.9, 95.0)

*Q1* 25th percentile, *Q3* 75th percentile*, FAST* Functional Assessment Staging Test, *Charlson* Charlson Comorbidity Index, *MMSE* Mini-Mental State Exam, *SDI* Sleep Disorder Inventory, *WASO* Wake After Sleep Onset

**Table 4:** Types of psychotropic medications prescribed for regular use at each time point

	Week 0 (baseline)		Week 8		Week 16		Week 24	
	Control (N = 36)	Intervention (N = 33)	Control (*N* = 28)	Intervention (*N* = 29)	Control (*N* = 21)	Intervention (*N* = 27)	Control (*N* = 24)	Intervention (*N* = 27)
Hypnotics and sedatives^a^	3 (8.3%)	6 (18.2%)	4 (14.3%)	4 (13.8%)	3 (14.3%)	4 (14.8%)	4 (16.7%)	2 (7.4%)
Benzodiazepines^b^	10 (27.8%)	13 (39.4%)	5 (17.9%)	13 (44.8%)	3 (14.3%)	12 (44.4%)	5 (20.8%)	9 (33.3%)
Antidepressants^c^	21 (58.3%)	16 (48.5%)	16 (57.1%)	11 (37.9%)	12 (57.1%)	11 (40.7%)	14 (58.3%)	11 (40.7%)
Antipsychotics^d^	20 (55.6%)	16 (48.5%)	17 (60.7%)	14 (48.3%)	12 (57.1%)	12 (44.4%)	12 (50.0%)	12 (44.4%)
Anti-dementia drugs^e^	7 (19.4%)	6 (18.2%)	5 (17.9%)	5 (17.2%)	2 (9.5%)	4 (14.8%)	4 (16.7%)	5 (18.5%)

Nmber of people who were prescribed each type of medication for regular use. ^a^Anatomical Therapeutic Chemical (ATC) classification N05C. ^b^ATC N05BA, benzodiazepine derivatives classified as anxiolytics. ^c^ATC N06A. ^d^ATC N05A. ^e^ATC N06D

**Table 5:** Outcome variables raw scores, all weeks. Median (Q1, Q3)

	Week 0 (baseline)		Week 8		Week 16		Week 24		
	Control (N = 36)	Intervention (N = 33)	Control (N = 28)	Intervention (N = 29)	Control (N = 21)	Intervention (N = 27)	Control (N = 24)	Intervention (N = 27)	Total (*N* = 225)
CSDD total	6.0 (4.0, 11.0)	11.0* (7.0, 14.0)	7.0 (4.0, 11.2)	10.0* (7.0, 13.0)	5.0 (1.0, 10.0)	6.0 (5.0, 9.0)	4.5 (1.8, 7.2)	8.0* (5.5, 12.5)	8.0 (4.0, 12.0)
CSDD Mood-related signs	2.0 (1.0, 3.0)	4.0* (2.0, 5.0)	2.5 (1.0, 4.0)	3.0 (2.0, 4.0)	2.0 (0.0, 4.0)	2.0 (1.0, 3.0)	2.0 (1.0, 3.0)	3.0* (2.0, 4.0)	3.0 (1.0, 4.0)
CSDD Behavioral disturbance	1.5 (0.8, 3.0)	2.0 (1.0, 4.0)	2.0 (1.0, 3.0)	3.0 (1.0, 4.0)	1.0 (0.0, 2.0)	2.0 (0.5, 3.0)	0.5 (0.0, 1.2)	3.0* (1.0, 4.0)	2.0 (1.0, 3.0)
CSDD Cyclic functions	1.0 (0.0, 2.0)	2.0 (0.0, 4.0)	2.0 (0.0, 3.0)	2.0 (1.0, 3.0)	0.0 (0.0, 2.0)	1.0 (0.0, 2.5)	1.0 (0.0, 2.0)	1.0 (0.0, 2.0)	1.0 (0.0, 2.0)
NPI-NH total	12.5 (5.8, 41.8)	24.0 (11.0, 42.0)	17.0 (5.8, 30.0)	19.0 (9.0, 34.0)	14.0 (6.0, 34.0)	13.0 (6.0, 26.2)	10.0 (5.0, 21.0)	20.0 (10.0, 28.0)	16.0 (6.0, 34.0)
NPI-NH Agitation	4.5 (0.0, 14.2)	6.0 (0.0, 12.0)	6.0 (2.0, 12.5)	4.0 (2.0, 14.0)	2.0(0.0, 14.0)	2.5 (0.2, 5.8)	4.0 (0.0, 8.0)	3.0 (1.0, 11.0)	4.0 (0.0, 12.0)
NPI-NH Affective symptoms	0.5 (0.0, 4.0)	2.0 (0.0, 10.0)	0.5 (0.0, 4.5)	1.0 (0.0, 6.0)	1.0 (0.0, 8.0)	1.0 (0.0, 4.0)	0.0 (0.0, 0.0)	2.0* (0.0, 6.0)	1.0 (0.0, 6.0)
NPI-NH Psychosis	0.0 (0.0, 8.0)	1.0 (0.0, 8.0)	0.0 (0.0, 3.0)	0.0 (0.0, 5.0)	0.0 (0.0, 4.0)	0.0 (0.0, 2.0)	0.0 (0.0, 4.0)	1.0 (0.0, 4.0)	0.0 (0.0, 6.0)

* p < 0.05, Kruskal-Wallis rank sum test for the difference between the control and intervention group. Q1 25th percentile, *Q3* 75th percentile, *CSDD* The Cornell Scale for Depression in Dementia, *NPI-NH* The Neuropsychiatric Inventory Nursing Home Version

### Light measurements

The mean vertical illuminance was 1039 lx (range 722–1242 lx) in the intervention condition and 242 lx (range 134–368 lx) in the control group. Mean CCT was 5369 K (range 5088–5641 K) in the intervention group and 3049 K (range 2707–3622 K) in the control group. In terms of melanopic EDI, the mean illuminance was 779 lx in the intervention group and 124 lx in the control group [45]. Although there was some variability between intervention units in the illuminance achieved, both illuminance and CCT were consistently higher in the intervention than in the control condition.

### The Cornell scale for depression in dementia

**Fig. 4:**
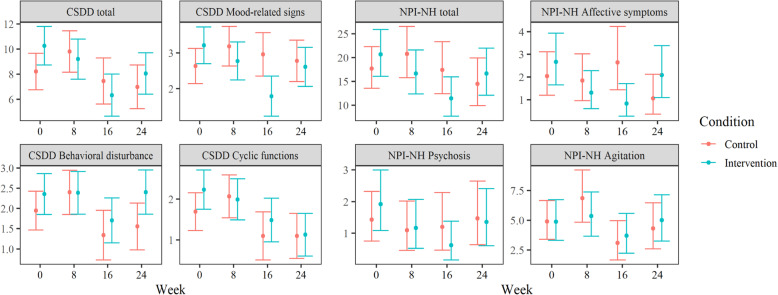
Estimated marginal means for all outcome measures by week. Scale of y-axis adapted to each outcome separately. Scaled for visibility, not reflective of the full range of possible scores. CSDD = The Cornell Scale for Depression in Dementia, NPI-NH = The Neuropsychiatric Inventory Nursing Home Version

Using a total score of 8 as a cut-off for depression on the CSDD [53], 14 patients in the control group and 24 in the intervention group could be categorized as clinically depressed at baseline. In week 16, this was reduced to 8 patients in the control group and 11 patients in the intervention group. Analyses of the total CSDD score (Table 6) found a significant interaction between week (baseline to week 16) and condition (B = − 3.2, 95% CI = − 6.0 – -0.3, *P* = 0.029) when controlling for the CSDD total at baseline and dementia severity (FAST score). The week by condition interaction indicates a difference between the control group and the intervention group in terms of change from baseline. This result therefore suggests that the intervention group had a larger change from baseline to week 16 than the control group. The estimated marginal mean (see Fig. 4), i.e., the mean adjusted for the influence of other variables in the model, was reduced from 10.3 (95% CI = 8.7–11.8) at baseline to 6.3 (95% CI = 4.6–8.0) at week 16 for the intervention group. In the control group, the estimated marginal mean was reduced from 8.2 (95% CI = 6.7–9.7) at baseline to 7.4 (95% CI = 5.6–9.3) at week 16. Thus, the CSDD total score was estimated to decrease with about 3.2 points (the coefficient for the interaction) more in the intervention group during the 16-week time span. When using FDR correction, this interaction failed to remain significant, indicating that there is a chance of this result being a false positive.

**Table 6:** Cornell Scale for Depression in Dementia predicted by week, condition, dementia severity and baseline total using multilevel regression

*Predictors*	Total		Mood-related signs		Behavioral disturbance		Cyclic functions	
	*Estimates (CI)*	*Std. betas (SD)*	*Estimates (CI)*	*Std. betas (SD)*	*Estimates (CI)*	*Std. betas (SD)*	*Estimates (CI)*	*Std. betas (SD)*
Week (8)	1.6 (− 0.3–3.5)	0.3 (1.6)	0.6 (− 0.1–1.2)	0.3 (1.8)	0.5 (− 0.2–1.1)	0.3 (1.3)	0.4 (− 0.3–1.0)	0.2 (1.1)
Week (16)	-0.8 (−2.8–1.3)	-0.1 (− 0.7)	0.3 (− 0.3–1.0)	0.2 (1.0)	− 0.6 (− 1.3–0.1)	− 0.4 (− 1.7)	− 0.6 (− 1.3–0.1)	− 0.3 (− 1.6)
Week (24)	− 1.2 (−3.2–0.8)	− 0.2 (− 1.2)	0.1 (− 0.5–0.8)	0.1 (0.5)	−0.4 (− 1.1–0.3)	−0.2 (− 1.1)	−0.6 (− 1.3–0.1)	−0.3 (− 1.7)
Condition [Intervention]	2.1 (− 0.1–4.2)	0.4 (1.9)	0.6 (− 0.1–1.3)	0.3 (1.6)	0.4 (− 0.3–1.1)	0.2 (1.1)	0.5 (−0.1–1.2)	0.3 (1.6)
*Interactions (indicating treatment effect)*								
Week (8) * [Intervention]	−2.7 (−5.4–0.0)	−0.5 (− 1.9)	− 1.0 ^*^(− 1.9 – − 0.1)	− 0.5 (− 2.3)	−0.4 (− 1.4–0.5)	−0.2 (− 0.9)	−0.6 (− 1.5–0.3)	−0.3 (− 1.3)
Week (16) * [Intervention]	− 3.2 ^*^(−6.0 – − 0.3)	− 0.6 (− 2.2)	*−1.8* ^***^(− 2.7 – − 0.8)	−0.9 (− 3.8)	−0.0 (− 1.0–0.9)	−0.0 (− 0.1)	−0.1 (− 1.1–0.8)	−0.1 (− 0.3)
Week (24) * [Intervention]	−1.0 (− 3.8–1.8)	−0.2 (− 0.7)	−0.8 (− 1.6–0.1)	−0.4 (− 1.7)	0.4 (− 0.5–1.4)	0.3 (0.9)	−0.5 (− 1.5–0.4)	−0.3 (− 1.1)
*Covariates*								
FAST	0.2 (−0.9–1.2)	0.0 (0.3)	−0.3 (− 0.6–0.1)	−0.1 (− 1.5)	0.1 (− 0.3–0.4)	0.0 (0.3)	0.2 (− 0.1–0.6)	0.1 (1.4)
Baseline DV	0.6 ***(0.4–0.7)	0.6 (8.6)	*0.6* ^***^(0.5–0.8)	0.7 (8.8)	*0.5* ^***^(0.4–0.6)	0.5 (7.4)	*0.5* ^***^(0.4–0.6)	0.6 (9.6)
*Model information*								
ICC (id)	0.25		0.32		0.18		0.11	
Marginal R^2^ / Conditional R^2^	0.423 / 0.567		0.436 / 0.614		0.334 / 0.454		0.415 / 0.480	

* p < 0.05, ** p < 0.01, *** p < 0.001. In italics: significant after Benjamini-Hochberg correction for false discovery rate with all eight models. Std. betas standardized regression coefficients, *SD* standard deviation, *CI* 95% confidence interval, *SD* standard deviation, *FAST* Functional Assessment Staging Test, *DV* dependent variable, *ICC* intraclass correlation coefficient

#### Sub-scale scores

The interaction between week and condition on the CSDD Mood-related signs was significant (*P* < 0.01) at week 16 with and without FDR correction (B = − 1.8, 95% CI = − 2.7 – − 0.8). This indicates that the intervention group scores were reduced with 1.8 points compared to the control group between baseline and week 16 when controlling for Mood-related signs at baseline and dementia severity (FAST score). The estimated marginal mean for Mood-related signs in the intervention group changed from 3.2 (95% CI = 2.7–3.7) at baseline to 1.8 (95% CI = 1.2–2.3) at week 16. In the control group, it was 2.6 at baseline (95% CI = 2.1–3.1) and 3.0 (95% CI = 2.4–3.6) at week 16. There were no significant interactions at weeks 8 or 24. There was no significant interaction in any week for the Behavioral disturbance or Cyclic functions sub-scales.

### The NPI-NH inventory nursing home version

The interaction between week and condition was significant at week 16 (B = − 1.1, 95% CI = − 2.2 – -0.1, *P* = 0.031, transformed scores) for the NPI-NH total score (Table 7) when controlling for the NPI-NH total score at baseline and dementia severity (FAST score). The estimated marginal mean (back-transformed) for the NPI-NH total score was 20.7 (95% CI = 16.0–25.9) at baseline and 11.4 (95% CI = 7.7–15.9) at week 16 for the intervention group. In the control group at week 16, it was 17.7 (95% CI = 13.6–22.3) at baseline and 17.4 (95% CI = 12.4–23.3). Thus, the estimated reduction in NPI-NH scores was 9.3 in the intervention group and near zero in the control group. With FDR correction, this interaction failed to reach significance, indicating that there is a possibility of this result being a false positive.

**Table 7:** Neuropsychiatric Inventory - Nursing Home Version (transformed scores) predicted by week, condition, dementia severity and baseline total using multilevel regression

*Predictors*	Total		Affective symptoms		Psychosis		Agitation	
	*Estimates (CI)*	*Std. betas (SD)*	*Estimates (CI)*	*Std. betas (SD)*	*Estimates (CI)*	*Std. betas (SD)*	*Estimates (CI)*	*Std. betas (SD)*
Week (8)	0.4 (− 0.3–1.0)	0.2 (1.0)	−0.1 (− 0.5–0.4)	−0.1 (− 0.3)	−0.1 (− 0.6–0.3)	−0.1 (− 0.7)	0.4 (− 0.1–0.9)	0.2 (1.6)
Week (16)	−0.0 (− 0.8–0.7)	−0.0 (− 0.1)	0.2 (− 0.3–0.7)	0.1 (0.8)	−0.1 (− 0.6–0.4)	−0.1 (− 0.4)	−0.5 (− 1.0–0.1)	−0.3 (− 1.7)
Week (24)	−0.4 (− 1.1–0.4)	−0.2 (− 1.0)	−0.4 (− 0.9–0.1)	−0.3 (− 1.6)	0.0 (− 0.5–0.5)	0.0 (0.1)	−0.1 (− 0.7–0.4)	−0.1 (− 0.5)
Condition [Intervention]	0.3 (− 0.4–1.1)	0.2 (0.9)	0.2 (− 0.3–0.7)	0.1 (0.8)	0.2 (−0.3–0.7)	0.1 (0.8)	−0.0 (− 0.5–0.5)	−0.0 (− 0.0)
*Interactions (indicating treatment effect)*								
Week (8) * [Intervention]	− 0.8 (− 1.8–0.1)	− 0.4 (− 1.7)	− 0.4 (− 1.1–0.2)	−0.3 (− 1.3)	−0.2 (− 0.8–0.4)	−0.1 (− 0.5)	−0.3 (− 1.0–0.4)	−0.2 (− 0.8)
Week (16) * [Intervention]	−1.1 ^*^(− 2.2 – − 0.1)	−0.5 (− 2.2)	*−0.9* ^**^(− 1.6 – − 0.2)	−0.7 (− 2.6)	−0.5 (− 1.2–0.1)	−0.4 (− 1.5)	0.2 (− 0.6–0.9)	0.1 (0.5)
Week (24) * [Intervention]	−0.1 (− 1.1–1.0)	−0.0 (− 0.1)	0.2 (− 0.5–0.9)	0.2 (0.6)	−0.2 (− 0.9–0.4)	−0.2 (− 0.7)	0.2 (− 0.6–0.9)	0.1 (0.5)
*Covariates*								
FAST	− 0.3 (− 0.7–0.1)	− 0.1 (− 1.6)	0.0 (−0.2–0.3)	0.0 (0.4)	−0.1 (− 0.3–0.1)	−0.0 (− 0.8)	−0.2 (− 0.5–0.1)	−0.1 (− 1.6)
Baseline DV	*0.7* ^***^(0.6–0.8)	0.7 (11.8)	*0.6* ^***^(0.5–0.7)	0.7 (12.0)	*0.6* ^***^(0.5–0.7)	0.7 (10.8)	*0.7* ^***^(0.6–0.9)	0.8 (14.3)
*Model information*								
ICC	0.24		0.56		0.60		0.70	
Marginal R^2^ / Conditional R^2^	0.516 / 0.630		0.037 / 0.580		0.019 / 0.610		0.013 / 0.701	

* p < 0.05, ** p < 0.01, *** p < 0.001. In italics: significant after Benjamini-Hochberg correction for false discovery rate) with all eight models. Std. betas standardized regression coefficients*,* SD standard deviation, *CI* 95% confidence interval, *SD* standard deviation, *FAST* Functional Assessment Staging Test, *DV* dependent variable, *ICC* intraclass correlation coefficient

#### Sub-syndrome scores

The interaction between week and condition on the Affective symptoms sub-syndrome was significant (*P* < 0.01) at week 16, both with and without FDR correction (B = − 0.9, 95% CI = − 1.6 – − 0.2, transformed scores) when controlling for Affective symptoms at baseline and dementia severity (FAST score). The estimated marginal mean (back-transformed) for Affective symptoms in the intervention group changed from 3.2 (95% CI = 1.7–5.1) at baseline to 1.1 (95% CI = 0.3–2.5) in week 16. In the control group, it was 1.6 at baseline (95% CI = 0.6–3.0) and 2.1 (95% CI = 0.8–4.0) in week 16. Thus, there was an estimated reduction of 2.1 points in the intervention group, and a slight increase of 0.5 points in the control group. There was no significant interaction at weeks 8 or 24. There was no significant interaction in any week for the sub-syndromes Psychosis or Agitation.

**Fig. 5:**
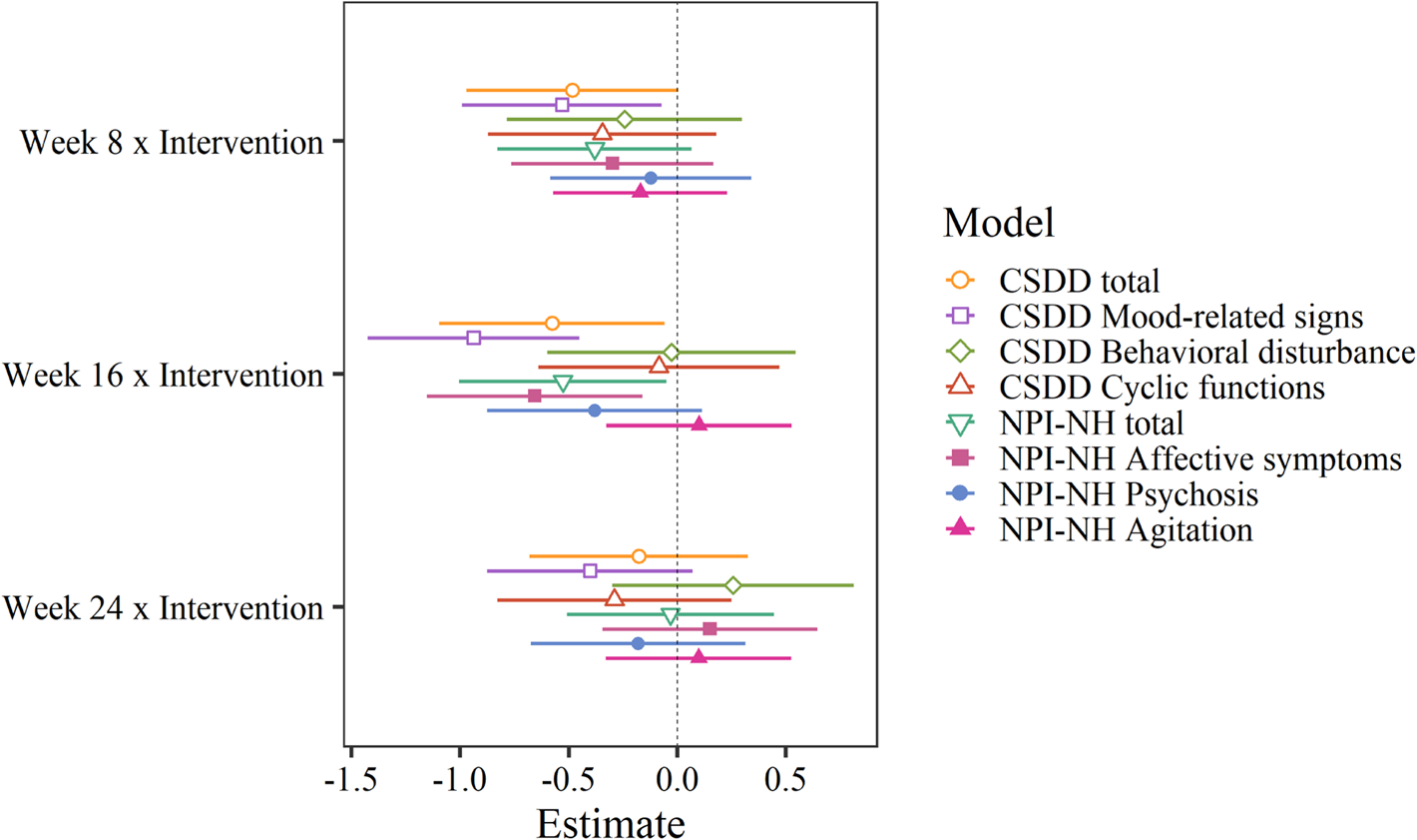
Standardized coefficients* and confidence intervals for the interaction between group (intervention vs. control) and time since baseline (week).*Predictors mean-centered, and dependent variable scaled. CSDD = The Cornell Scale for Depression in Dementia, NPI-NH = The Neuropsychiatric Inventory Nursing Home Version

A comparison of the standardized interaction coefficients for all BPSD outcome scales (indicating relative treatment effect) is shown in Fig. 5. When controlling for time spent in the living room, having an Alzheimer’s diagnosis, gender, eye disease, age, melanopic EDI, psychotropic medications, the use of sedatives or hypnotics, or score on the Charlson Comorbidity Index, coefficients remained the same or changed only marginally, not affecting significance levels. The only exception was that the Cornell total score coefficient for week 8 just reached significance (*p* = 0.047) before FDR correction with the addition of most covariates, indicating that this score was borderline significant before correcting for multiple measures. The sample size was not sufficient to perform sub-group analyses based on the variables controlled/adjusted for.

### Correlations with sleep and other outcome scales at baseline

**Fig. 6:**
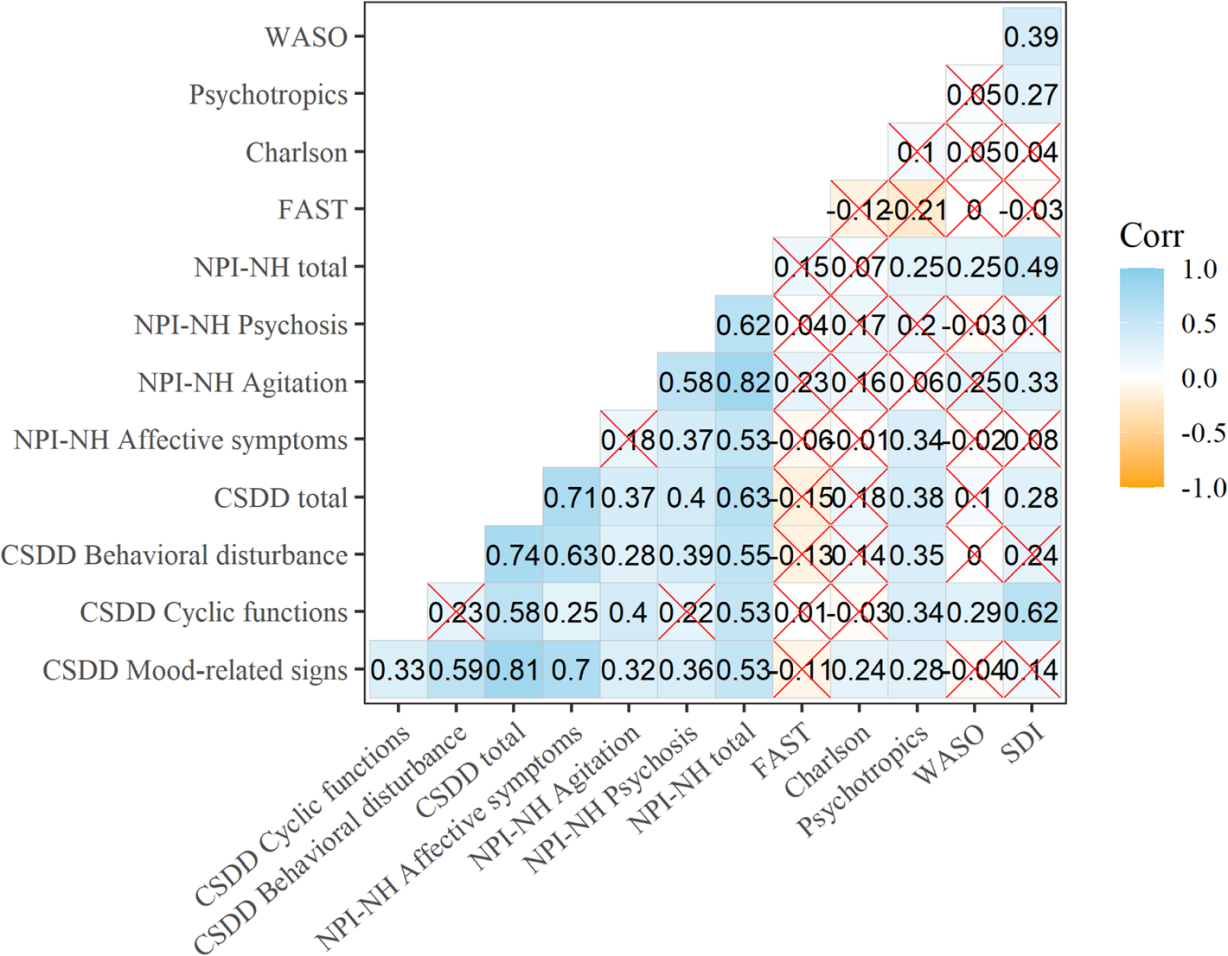
Spearman correlations at baseline. Non-significant (p > 0.05) correlations crossed out. CSDD = The Cornell Scale for Depression in Dementia, NPI-NH = The Neuropsychiatric Inventory Nursing Home Version

Spearman correlations at baseline are shown in Fig. 6. There was a significant correlation between the NPI-NH and CSDD total scores at baseline (rho = 0.63). CSDD Mood-related signs and NPI-NH Affective symptoms were highly correlated at 0.70, whereas NPI-NH Agitation and CSDD Behavioral disturbance only correlated at 0.28. WASO only correlated significantly with CSDD Cyclic functions (rho =0.29) and with the NPI-NH total (rho = 0.25), whereas the SDI total correlated with the CSDD Cyclic functions (rho = 0.62), the CSDD total (rho = 0.28), the NPI-NH Agitation (rho = 0.33), and NPI-NH total (rho = 0.49).

## Discussion

The present study provides some support for our hypothesis that BPSD can be improved by ceiling mounted BLT, specifically affective symptoms. Results showed significant improvements from baseline to week 16 in the intervention group as compared to the control group on the total scores of both the NPI-NH and CSDD, although not with false discovery rate correction. Only the NPI-NH Affective symptoms sub-scale and the CSDD Mood related signs showed significant group differences in change from baseline to week 16 after FDR correction. In short, the intervention group had an improvement in affective symptoms after 16 weeks of BLT compared to the control group. Our findings suggest that light has a potential clinical application in the management of mood related symptoms in people with dementia, with possible implications for the planning and design of dementia units.

The NPI-NH Affective symptoms and the CSDD Mood-related signs contain questions about depression and anxiety (see Table 2 for an overview of the scales). Although all items of the CSDD are designed to capture various symptoms of depression, mood-related symptoms may be less affected by difficulties with assessing ideation and somatic symptoms than the composite score. Reductions in expressions of affective symptoms, such as sadness and anxiety, are in line with previous research recommending BLT as an intervention for affective disorders [27–30]. However, the present findings diverge from a number of previous studies on BLT in dementia that have reported reduced agitation [10, 66, 67]. We could not replicate these findings using NPI-NH Agitation, or CSDD Behavioral disturbance scores. One reason for this discrepancy could be that previous studies have utilized different outcome measures for agitation [10, 66, 67].

Results on some sub-scales may have been impacted by the fact that certain symptoms are less common. The median scores on NPI-NH Psychosis were 0 in almost all weeks for both groups, and never above 1. Hence, detecting change on sub-syndromes or sub-scales comprising symptoms with very low frequency may require a larger sample size. In contrast, a relatively large number of patients had symptoms of depression, with 38 (55%) classified as depressed according to the CSDD.

The group difference in affective symptoms was only apparent at week 16, corresponding to the winter months of January/February. A possible explanation for this could be variations in availability of natural daylight. As week 24 occurred during springtime (April), week 16 represents the assessment point at which participants would have experienced the shortest period of daily natural illumination. This interpretation is in line with studies showing that seasonal affective disorder peak between December and February [68]. However, if the main effect of the BLT was to prevent deterioration during winter, we may have expected the control group to deteriorate, while the intervention group remained at pre-intervention levels. Rather, we found a reduction in scores from baseline to week 16 in the intervention group, and scores that either stayed the same or only slightly worsened in the control group.

The absence of an effect in week 8 could also indicate that the effects of BLT take a while to develop. This may be particularly true for patients suffering from severe dementia, because age and neurological disorders cause physiological changes that may affect circadian photoreception [69, 70]. A recent review reported that trials of at least 8 weeks appeared more effective at reducing depression and agitation in people with dementia compared to shorter trials [35]. It is also possible that the study did not have the statistical power to detect changes from baseline to week 8, as a non-significant reduction in the NPI-NH Affective symptoms and the CSDD Mood-related signs in the intervention group was seen already at week 8 in the present study.

A delay in effect would still not explain why symptoms return to pre-intervention levels in the intervention group at week 24. It may be that the onset of spring in week 24 introduced additional illumination both in the intervention area and in the patients’ bedrooms. Some researchers have raised concerns that excessive illumination may cause increased levels of agitation [38]. However, we did not find that scores on the NPI-NH Agitation or the CSDD Behavioral disturbance in the intervention group at week 24 were elevated above baseline levels.

Increased illumination in week 24 might have impacted BPSD indirectly by affecting circadian rhythms or sleep, but we found no significant change in daytime sleep or the total amount of sleep as measured by actigraphy as a result of the intervention [41]. Furthermore, the proxy-rated SDI showed significant improvements in sleep both at weeks 16 and 24 following BLT [41], indicating that caregiver perceptions of sleep problems did not increase prior to or at the same time as CSDD and NPI-NH assessed symptoms. At baseline, SDI correlated positively with the CSDD and NPI-NH totals but not with NPI-NH Affective symptoms or CSDD Mood-related signs. Taken together, this may suggest that the association between affective symptoms and measures of sleep are weak in this population, but research focusing on the relationship between these outcomes over time is needed. This is an issue that also needs to be addressed in future studies by controlling the light exposure from windows and other sources of artificial illumination outside of the main intervention area. Examination of individual variability in melatonin production and mid-winter activity levels would also be a valuable addition for exploration of why results varied over time.

The estimated group differences in change from baseline may have been inflated by the fact that scores on the CSDD and the NPI-NH were not equal between the conditions at baseline. The intervention group, with median score of 11 on the CSDD and 24 on the NPI-NH, had more room for improvement than the control group with a median score of 6 on the CSDD and 12.5 on the NPI-NH. Although baseline levels were included as predictors in the regression models, there is a possibility that changes in the intervention group could be attributed to a regression to the mean. The fact that group differences mainly resulted from symptom reduction in the intervention group, and not increased symptoms in the control group, further supports the notion that group differences at baseline influenced the present results.

Some studies on BLT have reported side-effects, although they are normally mild and transient [71]. We did not find evidence that any symptoms (including agitation) worsened during the 24-week period in the intervention group compared to the control group.

### Strengths and limitations of the study

The present study investigated short- and long-term effects of BLT, allowing for an investigation of both acute responses and of delayed effects. The 24-week time span of the trial exceeds most previous studies on BLT, allowing us to investigate the effects of BLT as well as the development of symptoms over time.

The participants represent a section of the population that is likely to experience a number of behavioral and psychological symptoms, but frequently excluded from trials due to the high occurrence of possible confounding factors such as multimorbidity and polypharmacy.

Using ceiling mounted light installations greatly reduces the demand on staff compared to the use of light boxes which require constant monitoring, and reduces the confounding impact such administration implies in terms of social interaction. Utilizing an intervention that conceivably could be implemented in dementia units also adds to the clinical relevance of the study. Resident medical practitioners and other staff were asked to continue treatment as usual, and daily routines were minimally disrupted, further adding to the ecological validity of the trial and the generalizability of the findings to clinical settings.

The use of ambient light installations also involves certain limitations. The intervention was not tailored to each individual, but rather provided a fixed schedule in terms of time and exposure. The optimal delivery of light treatment depends on individual circadian rhythms [72] and might therefore be more effective if timed according to each person’s sleep-wake rhythm. This would be a demanding approach, however, and may not be feasible in dementia units with limited available staff resources. Furthermore, the daily exposure time comprised a rough estimate. Still, the current design investigated the average effect of installing dynamic light fixtures under naturalistic conditions, albeit did not estimate the ideal duration of light exposure for each patient. Continuation of treatment as usual also involves the potential for confounding effects of psychopharmacological treatments, which could mask symptoms, lead to improvements independently of the BLT, or interact with the treatment effect. Investigating such an impact would require a considerably larger sample or a more selective screening of participants.

Inclusion mainly of very old and frail individuals with a high degree of cognitive impairment makes assessment of symptoms challenging. Our findings raise the possibility that treatment effect in this population might only be evident on questions relating to observable or overt symptoms. Future research with this population may thus consider utilizing assessment tools that to a larger extent evaluate observable behaviors. The rather small sample size is another limitation of the study. A larger sample size would have allowed for greater certainty regarding the reported results, and provided adequate power to perform subgroup-analyses, for instance by gender, depression scores at baseline, or dementia subtype. A larger number of clusters (nursing homes) would have allowed us to better account for the effect of clustering in regression models, and a larger sample of patients would be less vulnerable to group differences as baseline. Despite controlling for baseline scores in the analyses, differences in group scores at baseline raises questions about the internal validity of the present study, as the comparison group may not have provided an adequate control.

Furthermore, as light is a visible intervention, blinding of the staff to the condition assignment could not be achieved in the same way as with pharmacological trials. Although we strived to achieve a single blind design, some degree of response bias can therefore not be ruled out.

## Conclusions

The results of this 24-week trial indicate that ambient BLT may be effective at ameliorating affective symptoms after 16 weeks, but not after 8 or 24 weeks, among nursing home patients with dementia. The effects were especially evident on the CSDD Mood-related signs and NPI-NH Affective symptoms, which reflect observable signs of mood disorders such as sadness, crying, anhedonia and anxiety. This may indicate that BLT is effective mainly for affective symptoms during mid-winter in this population. There were no significant effects on other BPSD or sub-scales, and no indications of negative effects. We conclude that ambient BLT shows promise as a safe and non-invasive way to reduce affective symptoms, but future research is needed to determine why the effect was not observed after 8 or 24 weeks of BLT.

## Data Availability

The datasets generated and analyzed during the current study are not publicly available due to the risk of compromising the privacy of participating individuals but are available from the corresponding author on reasonable request.

## Abbreviations

BPSD: Behavioral and psychological symptoms of dementia
BLT: Bright light treatment
CCT: Correlated color temperature
NPI-NH: The Neuropsychiatric Inventory Nursing Home version
CSDD: The Cornell Scale for Depression in Dementia
MMSE: The Mini-Mental State Examination
FAST: The Functional Assessment Staging Test
SDI: The Sleep Disorder Inventory
WASO: Wake After Sleep Onset
FDR: False discovery rate
